# Age and blood pressure stratified healthy vascular aging, organ damage and prognosis in the community-dwelling elderly: insights from the North Shanghai Study

**DOI:** 10.1186/s40885-024-00288-3

**Published:** 2024-11-01

**Authors:** Zhongyuan Ren, Haotian Yang, Wenqing Zhu, Jun Han, Shikai Yu, Song Zhao, Weilun Meng, Yawei Xu, Yifan Zhao, Yi Zhang

**Affiliations:** 1 Heart Center, Shanghai Tenth People’s Hospital, School of Medicine, Tongji University, Shanghai, 200092 China; 2 School of Medicine, Tongji University, Shanghai, 200092 China; 3 Department of Cardiology, Rikaze People’s Hospital, Tibet, 857012 China

**Keywords:** Healthy vascular aging, Organ damage, Prognosis, Arterial stiffness, Elderly

## Abstract

**Background:**

This study aimed to investigate the prognostic value of age and blood pressure stratified healthy vascular aging (HVA) defined in the North Shanghai Study (NSS), and illustrate its relationship with organ damage (OD).

**Methods:**

This study enrolled 3590 community-dwelling elderly Chinese aged over 65 years and finally 3234 participants were included. 3230 individuals were included in the final analysis, with 4 participants lost to follow-up. NSS HVA was defined as low carotid-femoral pulse wave velocity (PWV) which had a higher cutoff value with advanced age and level of blood pressure. OD was thoroughly assessed and classified into vascular, cardiac and renal OD. Primary endpoints were major adverse cerebrocardiovascular events (MACCE) and all-cause mortality.

**Results:**

Nine hundred seventy-eight participants out of 3234 participants (43.1%) were identified as having NSS HVA. The NSS HVA group exhibited a younger age, lower blood pressure levels, lower body mass index, and milder OD compared to the non-NSS HVA group. Over follow-up of 5.7 ± 1.8 years, 332 MACCE (1.82 per 100 person-year) and 212 all-cause deaths (1.14 per 100 person -year) occurred. NSS HVA was associated with a reduced risk of MACCE (HR [95% CI] = 0.585, 0.454–0.754) and all-cause death (HR [95%CI] = 0.608 [0.445, 0.832]), especially in those subgroups without clinical diagnosed cardiovascular disease (CVD) or diabetes mellitus but with at least one type of OD. Moreover, NSS HVA exhibited improved prognostic value for MACCE, all-cause death and CVD death compared to other definitions of HVA.

**Conclusions:**

Age and blood pressure stratified NSS HVA could serve as an improved indicator against serious adverse events in the community-dwelling elderly Chinese.

**Trial registration:**

Prognosis in the Elderly Chinese: The Northern Shanghai Study (NSS), NCT02368938, https://clinicaltrials.gov/study/NCT02368938?cond=NCT02368938&rank=1.

**Supplementary Information:**

The online version contains supplementary material available at 10.1186/s40885-024-00288-3.

## Introduction

Healthy vascular aging (HVA), as defined by pulse wave velocity (PWV), characterizes a population exhibiting a relatively slow progression of vascular stiffness. The analysis from the Framingham Heart Study (FHS) initially established HVA in non-hypertensive individuals aged over 50, with PWV below the reference value of 7.6 m/s derived from a healthy young population [1]. However, due to the strong influence of age [2–4] and blood pressure [5] on PWV, the precise definition of HVA has been a subject of ongoing debate.

Recently, novel definitions of HVA have emerged, such as age-adjusted HVA proposed by Metabolic syndrome and Artery REsearch Consortium (MARE) [3] and our previous proposal of HVA stratified by both age and blood pressure in the North Shanghai Study (NSS HVA) [6]. Those definitions offer the potential to identify participants with a distinct higher prevalence of cardiovascular risk factors. Although these approaches hold promise, their effectiveness in risk stratification compared to classic FHS HVA requires in-depth investigation, particularly considering the limitations of cross-sectional study designs.

Preclinical organ damage (OD) refers to asymptomatic organ injury associated with blood pressure abnormalities. Research indicates that the presence of OD increases the risk of developing cardiovascular disease (CVD) and contributes to worse prognosis [7]. Consequently, several clinical guidelines recommend OD screening as an essential component of risk stratification for hypertensive patients [8].

As arterial stiffness plays a key role in the development and progression of hypertension [9], and PWV-derived indexes have demonstrated strong correlations with ODs [10–12]. However, the intricate interplay between vascular stiffness and OD has not been fully elucidated, and their relationship and impact on prognosis remain elusive.

This present study is based on data from the registered Northern Shanghai Study (NSS). The primary objectives are to observe the characteristics of age and blood pressure stratified definition of NSS HVA, explore the relationship between NSS HVA and OD, and investigate their respective roles in predicting adverse outcomes. The findings from this investigation may contribute valuable insights into risk assessment and management of cardiovascular disease in aging populations.

## Materials and methods

### Study population

**Fig. 1:**
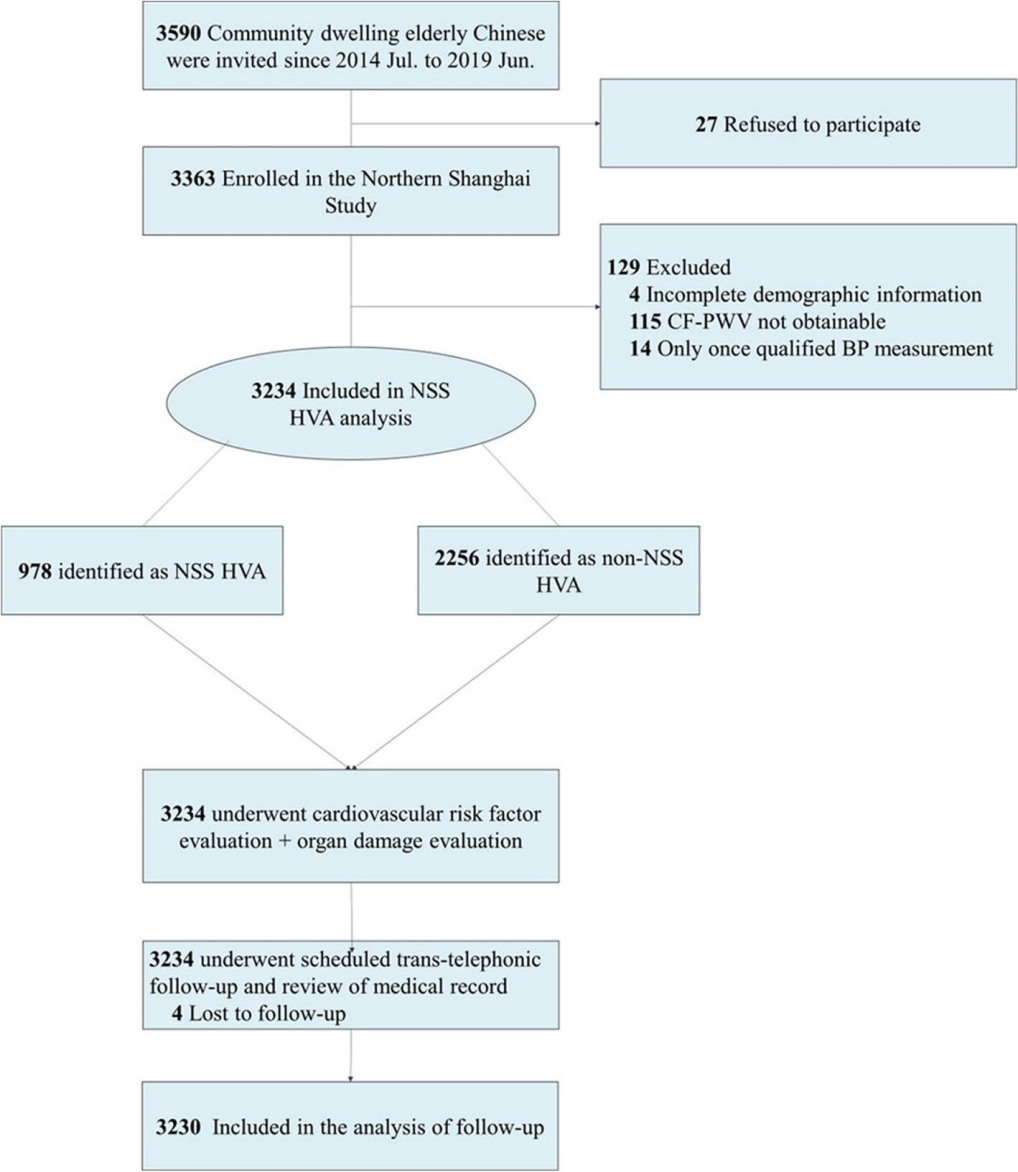
Study flow chart. Totally 3234 community dwelling Chinese elderly were included in the present study, and 3230 of them completed scheduled follow-up and included in the longitudinal analysis. BP denotes blood pressure, CF-PWV carotid femoral-pulse wave velocity, NSS HVA healthy vascular aging defined in the North Shanghai Study

A total of 3590 community-dwelling Chinese were invited, and 3363 of them were enrolled in the Northern Shanghai Study. Among them, 3234 participants had qualified data and were finally included in the present study of NSS HVA (the study flow chart is presented in Fig. 1). The Northern Shanghai Study is an ongoing prospective and registered study (ClinicalTrial.gov Identifier NCT02368938) and has been described in detail in our previous articles [13, 14]. The inclusion criteria of the Northern Shanghai Study were: 1) age ≥ 65 years; 2) local residents from urban communities in the Northern Shanghai, and 3) available for long-term follow-up. Meanwhile, the exclusion criteria were: 1) severe cardiac insufficiency (New York Heart Association functional class IV) or severe renal insufficiency (stage 5 chronic kidney disease); 2) history of stroke within 3 months; and 3) malignant tumor. Inform of consent was obtained from every participant. The study was approved by the ethics committee of Shanghai Tenth People’s Hospital.

### Data collection

Blood pressure was measured at the time of inclusion with standard criteria previously described [14]. Briefly, as participants sat upright, brachial blood pressure was measured using a mercury sphygmomanometer three times, with a five-minute interval between each measurement.The acquisition of medical history, anthropometrics, laboratory and imageological examination were detailed in Supplementary Files.

Primary endpoint is the composite of major adverse cerebrocardiovascular events (MACCE) which were defined as a composite of all-cause death, non-lethal myocardial infarction and non-lethal cerebral infarction and/or intracranial hemorrhage. CVD death was defined as death event directly resulted from recorded onset or exacerbation of CVD, including myocardial infarction, acute heart failure, malignant arrhythmia and cerebrovascular diseases. While non-CVD death was defined as death event resulted from diseases except for abovementioned CVD. Follow-up adverse events were obtained from medical records of National Healthcare Security Administration of China and Chinese Center for Disease Control and Prevention. All adverse events were diagnosed according to 10th edition of international classification code.

### Definition of NSS HVA

NSS HVA was defined based on the measurement of carotid-femoral PWV (CF-PWV). Different from classic HVA defined only by the cutoff value of CF-PWV (7.6 m/s) in Framingham study [1], the definition of NSS HVA in this study was based on CF-PWV stratified by age and blood pressure level, as previously reported [6]. Two levels of age, age 65–69 years and age ≥ 70 years, and three levels of blood pressure, < 120/80 mmHg, 120/80–130/85 mmHg and 130/85–140/90 mmHg were stratified, and higher CF-PWV cutoff values for NSS HVA were set with advanced age and higher blood pressure level. Furthermore, we also compared the predictive value of classic FHS HVA [1] and MARE HVA [3] (presented in Table 1).

**Table 1:** Cutoff value of CF-PWV for different definitions of HVA

**BP category (mmHg)**	**NSS HVA**		**FHS HVA**	**MARE HVA**
	**Age 65–69 yrs**	**Age ≥ 70 yrs**	**Age ≥ 50 yrs**	**age-quintile specific**
130/85—140/90	< 10.3 m/s	< 11.8 m/s	< mean CF-PWV + 2 SD of young (age < 30 years), non-hypertensive participants	< age-quintile specific 10th percentile of CF-PWV
120/80—130/85	9.7 m/s	11.7 m/s		
< 120/80	9.1 m/s	10.4 m/s		

*BP* denotes blood pressure, *CF-PWV* carotid-femoral pulse wave velocity, *HVA* healthy vascular aging

Blood pressure was measured with standard criteria at the time of inclusion. Participants who were on a hypertensive treatment would be categorized into non-HVA group. Participants would be categorized into corresponding blood pressure stratified reference levels if their measured blood pressure exceeded either systolic or diastolic lower limit

For CF-PWV, it was defined as pulse wave travel distance divided by the travel time. Two well-trained physicians recorded ipsilateral carotid and femoral pulse waves using applanation tonometry (SphygmoCor; AtCor Medical, Sydney, Australia). The superficial distances from the sternal notch to the right carotid artery and from the sternal notch to the right femoral artery were measured. The traveling distance of the pulse wave was calculated as the difference between the two measured distances. With pulse wave travel time automatically recorded, CF-PWV was automatically calculated by the device. Quality of measurement was guaranteed with an operator index > 80%.

### Evaluation of OD

Total six ODs were defined and classified according to the involved organ into cardiac, renal, and vascular ODs.

Cardiac ODs include left ventricular hypertrophy defined as left ventricular mass index (LVMI) ≥ 115 g/m^2^ for male, or ≥ 95 g/m^2^ for female, and left ventricular diastolic dysfunction (LVDD) defined as E/e’ ≥ 15 or 15 > E/e’ > 8, meanwhile, LVMI > 149 g/m^2^ for male or LVMI ≥ 122 g/m^2^ for female [15, 16].

Renal ODs include chronic kidney disease indicated by decreased estimated glomerular filtration rate (eGFR, ≤ 60 mL/min/1.73m^2^) and microalbuminuria indicated by increased urinary albumin-creatinine ratio (UACR) (≥ 30 mg/g).

Vascular ODs include impaired Ankle-brachial index (ABI, ABI ≤ 0.9), and carotid plaque was defined as the increment of carotid intima-media thickness (IMT) > 50% of the surrounding wall thickness or IMT > 1.5 mm on at least one side.

### Statistical analysis

Continuous data were presented as mean ± standard deviation if it conforms to normal distribution or median with interquartile range if it had a skewed distribution. Categorical data were presented as numbers with percentages. For comparison between two groups including NSS HVA and non-NSS HVA group, continuous data were compared using the student t-test if the data conformed to normal distribution with equal variance or the sign-rank test if the data presented skewed distribution and showed unequal variance. Chi-square test was applied for categorical variables. *P* for trend was calculated from logistic regression with the corresponding ordinal variables. Multivariate Logistic regression model for different OD of NSS HVA was adjusted for gender, smoke, alcohol, diabetes mellitus (DM) and CVD history.

Survival curves of all adverse events between groups were compared with a Kaplan–Meier estimate and a Log-Rank test. Further crude and adjusted Cox proportional hazard models were applied. Before Cox model, time-dependent Cox model, log(-log(survival)) plot and Schoenfeld residual plot were used to validate proportional hazards assumption. For adjusted Cox model 1, NSS HVA was adjusted for gender and age, and for adjusted model 2, NSS HVA was adjusted for gender, age, smoke, alcohol, DM and CVD history. For subgroup analysis, Cox proportional hazard model was applied to subgroups of different gender, BMI, with or without DM, with or without CVD history, and with or without concomitant OD. Predicting value among different definitions of HVA was compared using net reclassification index (NRI), integrated discrimination improvement (IDI) [17], and informativeness. Informativeness of different definitions of HVA was calculated as follows [18]:

$$\mathrm{Informativeness}=\frac{\chi^2\;\mathrm{for}\;\mathrm{interested}\;\mathrm{definition}\;\mathrm{of}\;\mathrm{HVA}}{\chi^2\;\mathrm{for}\;\mathrm{classic}\;\mathrm{HVA}}\left(\chi^2\;\mathrm{was}\;\mathrm{calculated}\;\mathrm{by}\;\mathrm{Wald}\;\mathrm{test}\;\mathrm{of}\;\mathrm{Cox}\;\mathrm{model}\right)$$

A two-tailed *P* value < 0.05 was considered significant. Statistical analysis system (SAS) version 9.4 (SAS Institute Inc.) was used for all the statistical analysis.

## Results

**Fig. 2:**
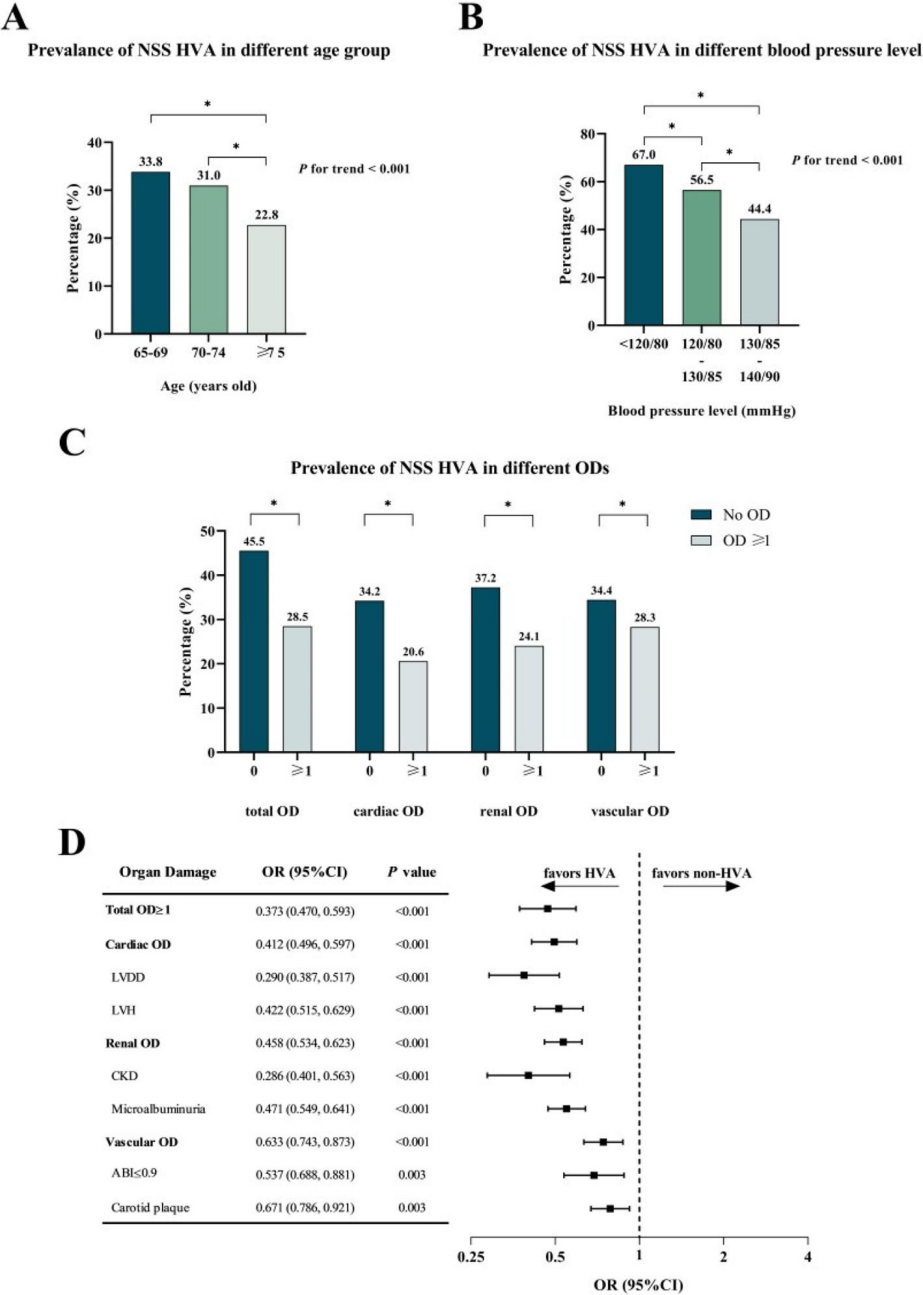
Part A-C prevalence of NSS HVA in different subgroups; Fig. 1 Part D, forest plot of multivariate Logistic regression for different OD of NSS HVA. **A** shows a decreasing prevalence of NSS HVA from younger to older age group; **B** shows a decreasing prevalence of NSS HVA from lower to higher blood pressure group; **C** shows prevalence of NSS HVA is distinctly higher in participants without OD, and similar results were observed in all specific types of ODs, indicating the strong correlation of NSS HVA and ODs; **D** Multivariate Logistic regression for different OD of NSS HVA that were adjusted for gender, smoke, alcohol, DM and CVD history. The forest plot illustrated that NSS HVA showed protective effect for all types of OD measured

Among 3234 community dwelling participants, NSS HVA was identified in nearly one third (*n* = 978) of the population. The included population had a lower proportion of male (*n* = 1395, 43.1%), while a similar gender proportion was observed between NSS HVA and non-NSS HVA group. The included population had a mean age of 71.4 ± 6.1 years, while participants with NSS HVA were significantly younger (70.4 ± 5.3 years in NSS HVA group vs 71.9 ± 6.3 years in non-NSS HVA group, *P* < 0.001). Though stratified by age and blood pressure, the prevalence of NSS HVA still presented a decreasing trend as the age or blood pressure rose, as shown in Fig. 2A and 2B. Besides, although the proportions of smokers and alcoholics similar, NSS HVA group showed significantly lower BMI and lower proportions of participants with DM, CVD, coronary heart disease and stroke. Most participants (*n* = 3018, 89.7%) were identified with at least one OD. Importantly, participants with NSS HVA had a distinctly lower prevalence of all types (cardiac, renal and vascular) of ODs, as depicted in Fig. 2C. Moreover, multivariate Logistic regression showed that NSS HVA was a strong protective factor for all types of ODs, as depicted in Fig. 2D. A detailed comparison of baseline information is listed in Table 2.

**Table 2:** Comparison of baseline characteristics in NSS HVA and non-NSS HVA group

**Clinical values**	**Overall (*n* = 3234)**	**NSS HVA (*n* = 978)**	**Non-NSS HVA (*n* = 2256)**	***P* value**
No. (%) of female	1839 (56.9)	557 (57.0)	1282 (56.8)	0.947
Age, median (IQR), years	70 (67–74)	69 (67–72)	70 (67–75)	< 0.001
BMI, median (IQR), kg/m^2^	24.0 (22.0- 26.0)	23.0 (21.0–25.0)	24.0 (22.0–26.0)	< 0.001
CF-PWV, mean (SD), m/s	9.0 (7.8–10.6)	7.9 (6.9–8.8)	9.6 (8.3–11.2)	< 0.001
Blood pressure, median (IQR), mmHg				
Systolic	135.3 (146.7–122.7)	122.0 (115.0- 130.0)	140.7 (130.0–150.7)	< 0.001
Diastolic	80.0 (72.0–85.3)	76.0 (70.0–80.0)	80.7 (75.0–88.7)	< 0.001
No. (%) of smoker	796 (24.6)	240 (24.5)	56 (24.7)	0.949
No. (%) of alcoholic	533 (16.5)	157 (16.1)	376 (16.7)	0.371
No. (%) with DM	628 (19.4)	120 (12.3)	508 (22.5)	< 0.001
No. (%) with CVD	1322 (40.9)	308 (31.5)	1014 (45.0)	< 0.001
No. (%) with CHD	997 (30.8)	230 (23.5)	767 (34.0)	< 0.001
No. (%) of smoker	588 (18.2)	120 (12.3)	468 (20.7)	< 0.001
No. (%) of alcoholic	699 (21.6)	199 (20.4)	500 (22.3)	0.244
**No. (%) with OD **^**a**^	2900 (89.7)	822 (84.1)	2078 (92.1)	< 0.001
**No. (%) with cardiac OD **^**a**^	937 (29.0)	190 (19.4)	747 (33.1)	< 0.001
LVMI, median (IQR), g/m^2^	83.2 (68.2–102.1)	76.6 (63.3–93.0)	86.3 (71.0–105.3)	< 0.001
No. (%) with LVH	764 (23.6)	155 (15.8)	609 (27.0)	< 0.001
E/e’, median (IQR)	8.8 (6.7–11.3)	8.0 (6.1, 10.5)	9.0 (7.0, 11.7)	< 0.001
No. (%) with LVDD	393 (12.2)	60 (6.1)	344 (15.2)	< 0.001
**No. (%) with renal OD **^**a**^	1702 (52.6)	406 (41.5)	1296 (57.5)	< 0.001
eGFR, median (IQR), ml/(min*1.73m^2^)	84.3 (74.1–96.1)	86.6 (77.7–97.3)	83.1 (72.5–95.1)	< 0.001
No. (%) with CKD	278 (8.6)	42 (4.3)	236 (10.5)	< 0.001
UACR, median (IQR), mg/g	30.4 (14.3–58.5)	23.5 (11.2–44.6)	34.6 (16.2–65.7)	< 0.001
No. (%) with microalbuminuria	1604 (49.6)	382 (39.1)	1222 (54.2)	< 0.001
**No. (%) with vascular OD **^**a**^	2220 (68.7)	623 (63.7)	1597 (70.8)	< 0.001
Average ABI, median (IQR)	1.07 (1.00–1.14)	1.08 (0.93–1.14)	1.07 (0.99–1.14)	0.003
No. (%) with ABI ≤ 0.9	409 (12.7)	93 (9.5)	316 (14.0)	< 0.001
Average IMT, median (IQR), mm	0.64 (0.54–0.76)	0.61 (0.52–0.74)	0.65 (0.55–0.77)	< 0.001
No. (%) with carotid plaque	2129 (65.8)	603 (61.7)	1526 (67.6)	0.004

Continuous variables are presented as mean with standard deviation; categorical variables are presented as number with percentage

*ABI* ankle-brachial index, *BMI* body mass index, *CF-PWV* carotid-femoral pulse wave velocity, *CHD* coronary heart disease, *CKD* chronic kidney disease, *CVD* cardiovascular disease, *DM* diabetes mellitus, *eGFR* estimated glomerular filtration rate, *E/e’* the ratio of early diastolic mitral inflow velocity to early diastolic mitral annulus velocity, *HVA* healthy vascular aging, *IMT* intima media thickness, *LVDD* left ventricular diastolic dysfunction, *LVH* left ventricular hypertrophy, *LVMI* left ventricular mass index, *UACR* urinary albumin-creatinine ratio

^a^Number of OD listed here indicate participants with the presence of at least one corresponding OD (s)

**Fig. 3:**
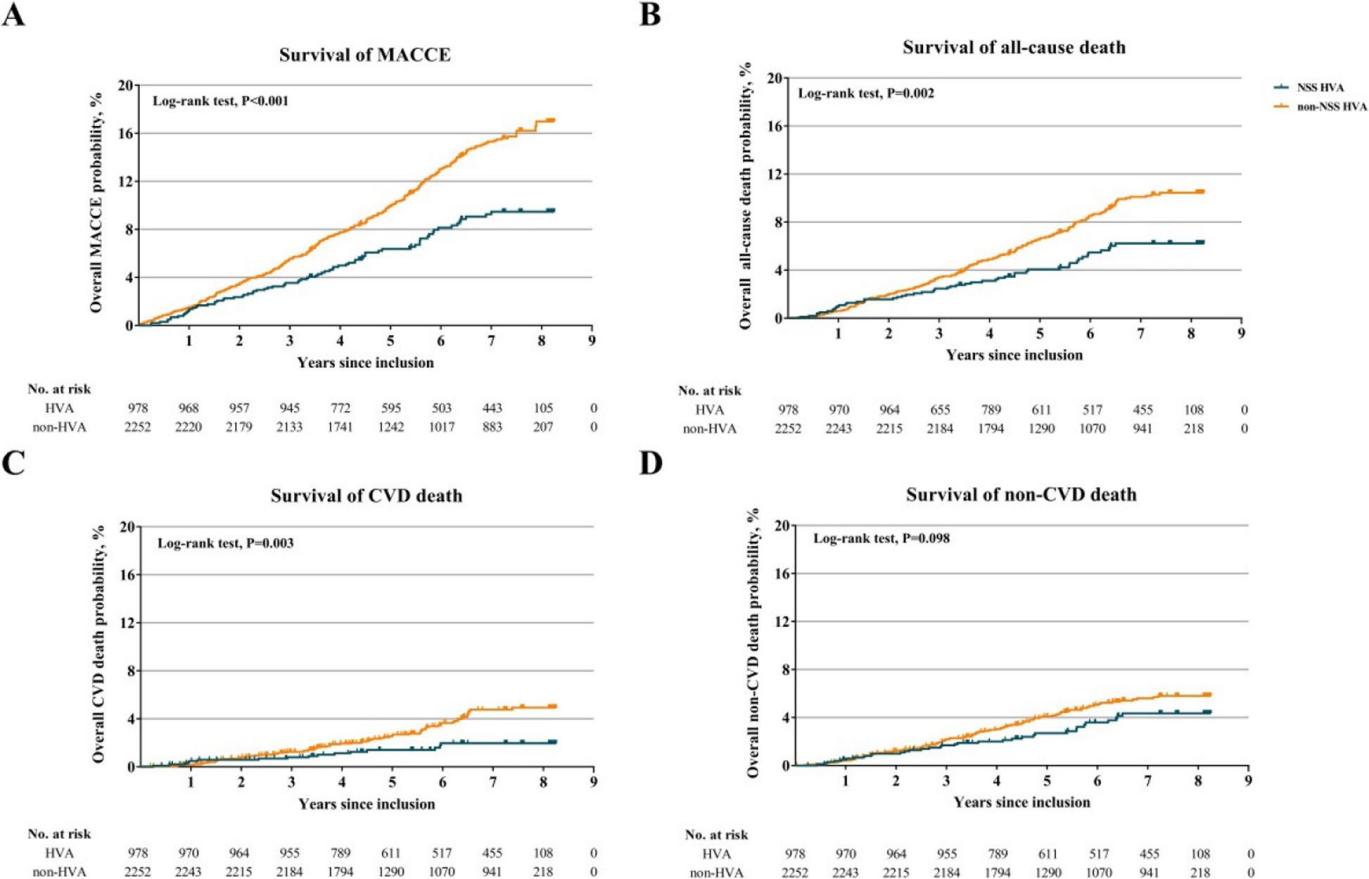
Part A-D Survival curves of MACCE, all-cause death, CVD and non-CVD death. Compared with participants without NSS HVA, participants with NSS HVA presented significantly higher survival for MACCE (A), all-cause death (B) and CVD death (C). While the difference in survival of non-CVD is not significant. CVD denotes cardiovascular disease, MACCE major adverse cardiocerebrovascular events

Through a follow-up of 5.7 ± 1.8 years, 4 participants (0.1%) were lost to follow-up. Totally 332 MACCEs were observed, 64 cases in the NSS HVA group, and 268 in the non-NSS HVA group. Survival analysis showed that incidences of MACCE, all-cause death and CVD death were significantly lower in the NSS HVA group, as depicted in Fig. 3. While incidences of death due to non-CVD and intracranial hemorrhage events were comparable. Besides, the crude and adjusted Cox proportional hazard model showed NSS HVA was a protective factor for MACCE, all-cause death, and other events except non-CVD death and intracranial hemorrhage, as listed in Table 3.

**Table 3:** Incidences of adverse events and results of Cox proportional hazard model

	**Incidence (per 100 person-year)**				**Crude Cox model**		Adjusted Cox model 1 ^*^		**Adjusted Cox model 2 **†	
	**Overall (*n* = 3230)**	**NSS HVA (*n* = 978)**	**Non-NSS HVA (*n* = 2252)**	***P*-value**	**HR (95%CI)**	***P*-value**	**HR (95%CI)**	***P*-value**	**HR (95%CI)**	***P*-value**
**MACCE**	332 (1.82)	64 (1.13)	268 (2.14)	< 0.001	0.585 (0.454–0.753)	< 0.001	0.671 (0.546- 0.825)	< 0.001	0.585 (0.454- 0.754)	< 0.001
**All-cause death**	212 (1.14)	41 (0.71)	171 (1.33)	0.003	0.608 (0.444- 0.831)	0.002	0.610 (0.446- 0.834)	0.002	0.608 (0.445- 0.832)	0.002
CVD death	42 (0.23)	6 (0.10)	36 (0.28)	0.005	0.454 (0.265- 0.777)	0.004	0.455 (0.266- 0.779)	0.004	0.453 (0.264- 0.775)	0.004
Non-CVD death	103 (0.55)	20 (0.35)	83 (0.65)	0.132	0.722 (0.491- 1.064)	0.100	0.726 (0.493- 1.068)	0.104	0.726 (0.492- 1.067)	0.103
**Myocardial Infarction **‡	88 (0.47)	18 (0.31)	70 (0.55)	0.024	0.366 (0.154- 0.869)	0.023	0.024 (0.155- 0.875)	0.024	-	-
**Stroke**	15 (0.08)	2 (0.03)	13 (0.10)	0.023	0.571 (0.361- 0.902)	0.017	0.572 (0.361- 0.904)	0.017	0.569 (0.360- 0.901)	0.016
Cerebral infarction	212 (1.14)	41 (0.71)	171 (1.33)	0.028	0.552 (0.334- 0.912)	0.021	0.553 (0.335- 0.915)	0.021	0.550 (0.333- 0.910)	0.020
Intracranial hemorrhage	42 (0.23)	6 (0.10)	36 (0.28)	0.547	0.684 (0.223- 2.098)	0.507	0.684 (0.223- 2.097)	0.506	-	-

^*^Model 1 was adjusted for gender

†Model 2 was adjusted for gender, smoker, alcoholic, DM and CVD history

‡In consideration of overfitting, the events amount of myocardial infarction and intracranial hemorrhage are too small to be adjusted for five variables in model 2 CVD denote cardiovascular disease, HVA healthy vascular aging, MACCE major cardiocerebrovascular events, HR hazard ratio, 95%CI, 95% confidence interval

**Fig. 4:**
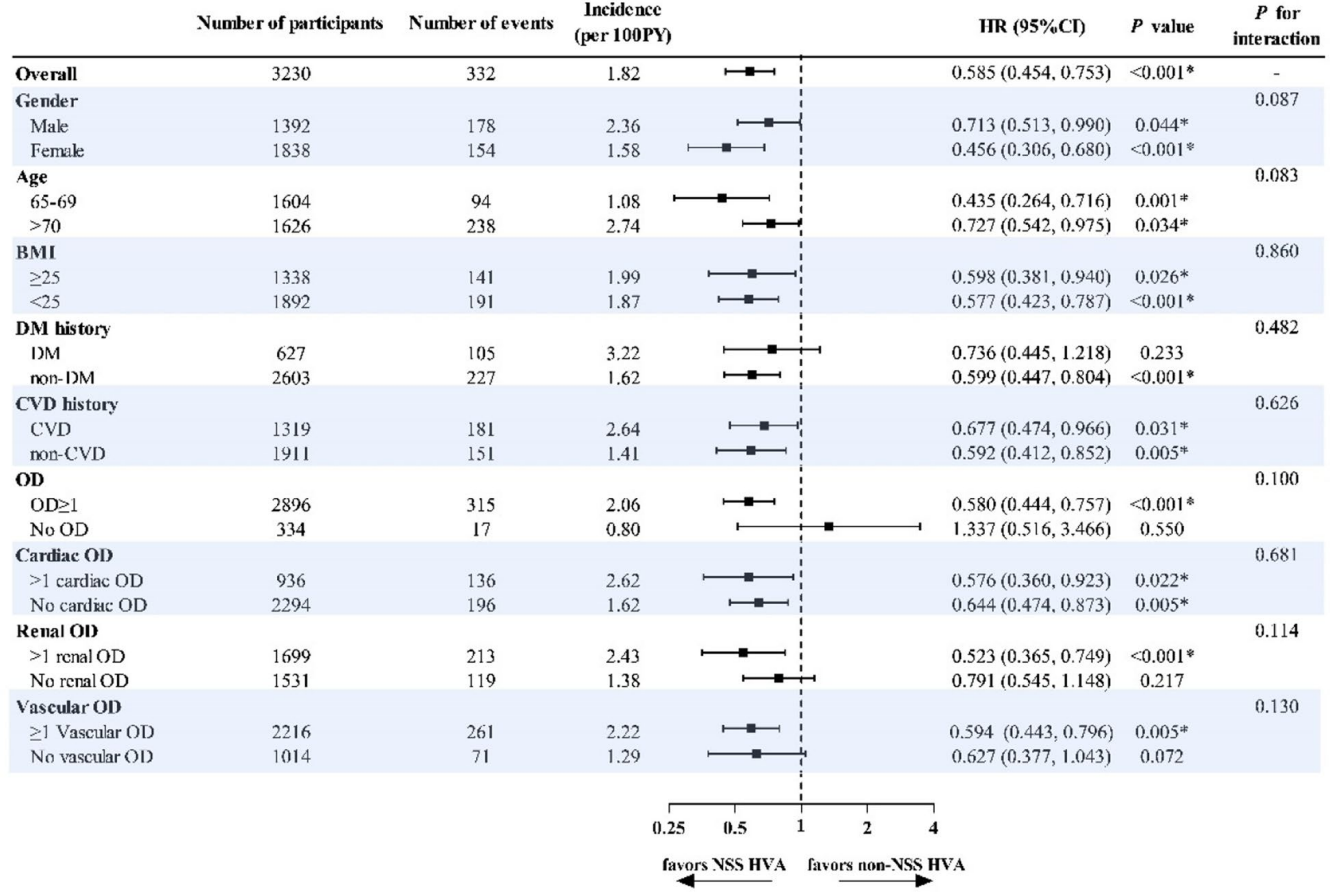
Forest plot of subgroup analysis for MACCE. HVA showed protective value in all subgroups except in participants with DM history, without any OD, without renal OD and without vascular OD. BMI denotes body mass index, CVD cardiovascular disease, DM diabetes mellitus, OD target organ damage, PY person-year, * indicates significant *P* value < 0.05

Subgroup analysis was performed to investigate the protective value of NSS HVA further. For MACCE (illustrated in Fig. 4), no interaction of NSS HVA with the listed variables was found. NSS HVA showed homogeneously significant protective value except in participants with a history DM, without any OD and especially without renal and vascular OD. While the predictive value of NSS HVA for all-cause death was also homogeneously significant except in subgroups of females, age ≥ 70 years, BMI < 25 kg/m^2^, with DM history, with CVD history, with cardiac history, without renal and vascular OD (Supplementary Fig. 1).

Furthermore, we compared the predictive ability of three definitions of HVA, including FHS HVA, MARE HVA, and NSS HVA. Compared with FHS HVA, NSS HVA presented a positive NRI and IDI despite not statistically significant. And NSS HVA provided 148% higher informativeness for MACCE and 263% higher informativeness compared with FHS HVA, as presented in Table 4.

**Table 4:** Comparison of predictive ability of FHS HVA, MARE HVA and NSS HVA

	**Crude COX model**		**NRI**		**IDI**		**Informativeness**	
	**HR (95%CI)**	***P* value**	**NRI (95%CI)**	***P* value**	**IDI (95%CI)**	***P* value**	**χ**^**2**^** statistics**	**Relative informative-ness**
**MACCE**								
FHS HVA	0.407 (0.256–0.646)	< 0.001	Ref	-	Ref	-	14.5	100%
MARE HVA	0.760 (0.498–1.161)	0.205	-0.158 (-0.214–0.102)	0.006	-0.005 (-0.006–0.003)	< 0.001	1.6	11.1%
NSS HVA	0.585 (0.454–0.753)	< 0.001	0.087 (0.008–0.166)	0.133	0.002 (-0.001–0.004)	0.204	21.5	148%
**All-cause death**								
FHS HVA	0.559 (0.336–0.931)	0.025	Ref	-	Ref	-	5.0	100%
MARE HVA	0.904 (0.551–1.484)	0.690	-0.112 (-0.188–0.038)	0.113	-0.002 (-0.003–0.001)	0.002	0.2	3.2%
NSS HVA	0.608 (0.444–0.831)	0.002	0.120 (0.029–0.212)	0.090	0.002 (0.001–0.004)	0.005	13.1	263%
**CVD death**								
FHS HVA	0.362 (0.132–0.988)	0.047	Ref	-	Ref	-	3.9	100%
MARE HVA	1.167 (0.562–2.422)	0.680	-0.133 (-0.267- 0.001)	0.240	0.006 (-0.002–0.001)	0.006	0.2	4.3%
NSS HVA	0.454 (0.265–0.777)	0.004	0.178 (0.052–0.305)	0.116	0.002 (0.001–0.003)	0.003	10.4	264%

*FHS* denotes Framingham Heart Study, *MARE* Metabolic syndrome and Artery REsearch Consortium, *NSS* the North Shanghai Study, *NRI* net reclassification index, *IDI* integrated discrimination improvement

## Discussion

The present study represented a community-based longitudinal investigation that meticulously examined the characteristics of age and blood pressure stratified NSS HVA and assessed its prognostic significance. The major findings were: 1. Participants with NSS HVA had better conditions, with lower prevalence of subclinical ODs; 2. Participants with NSS HVA presented better prognoses of MACCE, all-cause mortality and cardiovascular mortality; 3. The predictive value of age and blood pressure stratified NSS HVA outperforms classic FHS HVA and MARE HVA.

### Identifying NSS-HVA and its prognostic value of MACCE

CF-PWV has been recognized as a marker of vascular aging and has been shown to be associated with cardiovascular disease outcomes. However, CF-PWV is significantly influenced by age and blood pressure, making it unable to accurately distinguish between HVA and accelerated vascular aging when these factors are not considered. Using only a cutoff value of CF-PWV to define HVA without accounting for the effects of blood pressure and age is not particularly accurate. Therefore, a more precise definition of HVA is especially important.

The definition of HVA has been a subject of prolonged debate. The FHS analysis adopted a simplistic and convenient approach, defining HVA as non-hypertensive individuals aged over 50 with a PWV less than 7.6 m/s (equivalent to the mean + 2 standard deviations of PWV in healthy individuals under 30 years of age) [1]. However, this single cutoff value overlooks the impact of advancing age on PWV progression. Research has revealed that blood vessels naturally undergo degeneration with age, with arteries stiffening at an average speed of 0.2–0.7 m/s PWV per 5 years [2]. Consequently, the prevalence of FHS HVA decreases steeply in older age groups, with only 1.0% (6 participants) out of 617 participants being recognized as having FHS HVA in advanced age [1]. Therefore, defining HVA with age stratification may provide better identification of HVA in various age cohorts. A pooled population study involving 18,490 participants from the Study of Metabolic Syndrome and Artery Research Consortium proposed an age-specific definition of HVA. This definition determined HVA based on the cutoff of the lowest 10% of PWV in each quintile age group. This age-specific definition identified more participants with MARE HVA in older age groups, and these individuals exhibited significantly fewer cardiometabolic risk factors [3]. However, due to the cross-sectional design of their study, the prognostic value of this age-specific definition was not investigated.

In addition to age, blood pressure also plays a crucial role in vascular aging. Although the relationship between blood pressure and vascular stiffness may be bidirectional, where elevated blood pressure could worsen arterial stiffness [5] and vice versa. Research has confirmed that individuals with higher blood pressure tend to have correspondingly higher PWV values [1, 3]. Previous research from the Framingham Study excluded participants with hypertension or those on hypertensive medication when defining HVA. However, the PWV cutoff value was not stratified by blood pressure in participants with normal or high-normal blood pressure. Their results showed that the average blood pressure was significantly lower in the HVA group (113 ± 11 mmHg for HVA vs. 130 ± 18 mmHg for No HVA), and the predictive value of HVA was compromised after adjusting for systolic blood pressure, highlighting the significant contribution of blood pressure to HVA [1]. Considering the importance of age and blood pressure in vascular aging, our study aimed to establish an age and blood pressure stratified definition of HVA. Our findings identified more participants with HVA, particularly over 20% of HVA in the elderly population aged over 70 years. Importantly, these HVA participants not only exhibited significantly fewer cardiovascular risk factors [6], but also had notably better survival rates concerning MACCE and all-cause mortality, both in young and aged subgroups. Based on our results, age and blood pressure stratified NSS HVA could serve as a valuable prognostic indicator in the community dwelling Chinese population.

When comparing the predictive value of three different HVA definitions in our study population, we found that age and blood pressure stratified NSS HVA demonstrated superior informativeness and better identification of patients at risk for MACCE. It is reasonable due to our stratification of both age and blood pressure which are two most important influence factors for PWV, especially in elderly population. Nevertheless, it is crucial to recognize that the definition of HVA may be specific to different racial or ethnic populations. As our comparison was limited to a community-dwelling elderly Chinese population, further large-scale, longitudinal studies are warranted to evaluate different HVA definitions across diverse populations. Such research will provide comprehensive insights into the optimal HVA definition and its clinical utility for risk assessment and management.

### HVA and OD

A substantial body of evidence has been published highlighting the close relationship between arterial stiffness and OD [10, 11]. However, despite the prevalence of cross-sectional study designs in most of these investigations, the impact of the interplay between arterial stiffness and OD on prognosis remains insufficiently explored. A recent study by Vasan et al. sought to shed light on this interrelation by analyzing data from 5,803 participants in the Framingham Heart Study [11]. They revealed that arterial stiffness partially mediated the association between OD and incident CVD. Their findings demonstrated that the prognostic value of OD was attenuated by 5–21% after adjusting for arterial stiffness. In line with this research, our study aimed to explore the relationship between HVA and OD from a different perspective. We observed a strong correlation between vascular aging and OD, as evidenced by a significantly lower prevalence of OD in participants with NSS HVA and vice versa—a significantly lower prevalence of NSS HVA in participants with any form of OD. Since OD usually reflects a subclinical pathophysiological condition leading to an increased incidence of adverse cardio-cerebral vascular events [19], our findings indicated NSS-HVA may predict incident adverse cardio-cerebral vascular events in an early phase and providing evidence to support early treatment for avoiding MACCE.

However, it is essential to note that the protective value of NSS HVA was compromised in two specific populations. Firstly, in very healthy individuals without any OD, the overall incidence of severe adverse events was remarkably low, making it challenging to discern distinct differences between the NSS HVA and non-NSS HVA groups. Secondly, in populations with diagnosed diseases such as DM, the protective effect of NSS HVA was also diminished. In such cases, the development of DM can be outcomes of arterial stiffness and/or OD progression [20–22]. While arterial stiffness might still play a role in determining prognosis, the ultimate outcome could be more influenced by the secondary prevention and management of the specific disease. The predictive value of NSS HVA in populations with diagnosed DM necessitates further investigation in comparable patients with optimized treatment strategies. Based on the current evidence, we cautiously conclude that NSS HVA could serve as a protective indicator in populations with existing OD. However, for populations free from OD and those with diagnosed DM, additional studies are warranted to draw conclusive insights into the prognostic significance of NSS HVA in these specific scenarios.

### Limitation

Caution should be exercised when interpreting our findings. Firstly, generalizing our results to other populations, including younger individuals, diseased populations, and individuals of different racial backgrounds, should be done with care, as our study focused on a specific community-dwelling elderly Chinese cohort. Secondly, although we comprehensively assessed three types of ODs using two indices each, our OD assessment may not cover all possible manifestations. For instance, brain OD, including lacunar infarction and cognitive assessment, was not included in our evaluation. Additionally, microvascular obstruction (in the eyes or coronary arteries) could represent a novel indicator of OD, necessitating future studies with more comprehensive OD assessments. Furthermore, our study primarily focused on MACCE and mortality without evaluating the progression of OD during the follow-up period. To fully understand the interrelation between vascular stiffness and OD, well-designed longitudinal studies with comprehensive OD evaluations are required.

## Conclusion

Age and blood pressure stratified NSS HVA emerged as a valuable protective factor against serious adverse events in the context of community-dwelling elderly Chinese individuals. However, further validation is needed to determine the protective value of NSS HVA in specific populations.

## Supplementary Information

Supplementary Material 1.

## Data Availability

The data and materials utilized in this research are available upon request. Researchers interested in accessing the data and materials for further analysis or replication purposes can contact [Haotian Yang/yht201400@163.com] for assistance. We believe in fostering transparency and reproducibility in scientific endeavors, and thus, we are committed to making our research materials accessible to the broader academic community.

## Abbreviations

HVA: Healthy vascular aging
PWV: Pulse wave velocity
FHS: Framingham Heart Study
MARE: Metabolic syndrome and Artery REsearch Consortium
NSS: North Shanghai Study
OD: Organ damage
CVD: Cardiovascular disease
CF-PWV: Carotid-femoral PWV
LVMI: Left ventricular mass index
LVDD: Left ventricular diastolic dysfunction
Egfr: Estimated glomerular filtration rate
UACR: Urinary albumin-creatinine ratio
ABI: Ankle-brachial index
IMT: Intima-media thickness
DM: Diabetes mellitus
NRI: Net reclassification index
IDI: Integrated discrimination improvement
