# Supplementary material for: Age and blood pressure stratified healthy vascular aging, organ damage and prognosis in the community-dwelling elderly: insights from the North Shanghai Study
Source: Clin Hypertens. 2024 Nov 1;30:31. doi: 10.1186/s40885-024-00288-3 (PMC11529181; doi:10.1186/s40885-024-00288-3)
Supplement: Supplementary file 1 — Supplementary Material 1. [file 40885_2024_288_MOESM1_ESM.docx]

**Supplementary files**

# Methods

**Medical history collection**

An organized questionnaire was used to collect the medical history and familial history of each participant, encompassing present illness, smoking status, and history pre-existing diseases such as hypertension, DM, and cardiovascular, cerebrovascular and renal diseases. Diagnosis of aforementioned previous illness was confirmed with participants whose corresponding medical record was available and could be traced. All the information was collected by well-trained physicians at community healthcare center before any measurement.

**Blood pressure measurement**

With height and weight measured, body mass index (BMI) was calculated as weight divided by squared height. BP was measured by well-trained physicians in a room with standard temperature (22-27℃). Participants were required to sit upright when brachial BP was measured by a mercury sphygmomanometer. Totally each participant was measured for 3 times, with the interval of 5 minutes. Hypertension was defined as measured average systolic BP ≥ 140mmHg or diastolic BP ≥ 90 mmHg or taking any antihypertensive agents.

**Biochemical sample collection**

Blood and urine samples of each participant were analyzed in the Department of Laboratory Medicine of Shanghai Tenth Peoples’ Hospital by experienced technicians. Blood and urine routine tests were conducted, and biochemical measurement of blood glucose, glycated hemoglobin, lipid metabolism markers, blood and urinary creatinine, and urinary albumin excretion were examined. eGFR was calculated according to the modified MDRD (modification of diet in renal disease) formula: eGFR (mL/min/1.73 m2) =175×Cr−1.234 × age−0.179 (women ×0.79). While UACR was calculated as urinary microalbumin level divided by urinary creatinine level.

**Echocardiography collection**

Transthoracic echocardiography was performed by two experienced physicians using MyLab 30 CB (ESAOTE SPA). In the parasternal long-axis view, left ventricular end-diastolic diameter (LVEDd), interventricular septal diameter (IVSd), posterior wall thickness end-diastole diameter (PWTd) and left ventricular end-systolic diameter (LVESd) were indices measured, which is in accordance with the guidelines of American Society of Echocardiography (ASE) (19).

Left ventricular mass index (LVMI) = (0.8×1.04× ((LVEDd+PWTd+IVSd)^3^−(LVEDd)^3^) +0.6) / (body surface area). left ventricular hypertrophy was defined as left ventricular mass index (LVMI) ≥115 g/m^2^ for male, or ≥95 g/m^2^ for female.

E/e’, the ratio of peak trans-mitral flow velocity and peak lateral tricuspid velocity at early diastole. left ventricular diastolic dysfunction (LVDD) defined as E/e’≥15 or 15＞E/e’＞8 mean while LVMI＞149 g/m^2^ for male or LVMI≥122 g/m^2^ for female.

**Vascular measurement**

Ankle-brachial index (ABI) was defined as the brachial systolic BP divided by ankle systolic BP that measured by the VP1000 system (Omron, Tokyo, Japan). Bilateral ABI was measured and calculated concurrently. Bilateral carotid plague and carotid intima-media thickness (IMT) were evaluated by ultrasonography using MyLab 30 Gold CV system (ESAOTE SpA, Genoa, Italy).

# Figures and figure legends

**
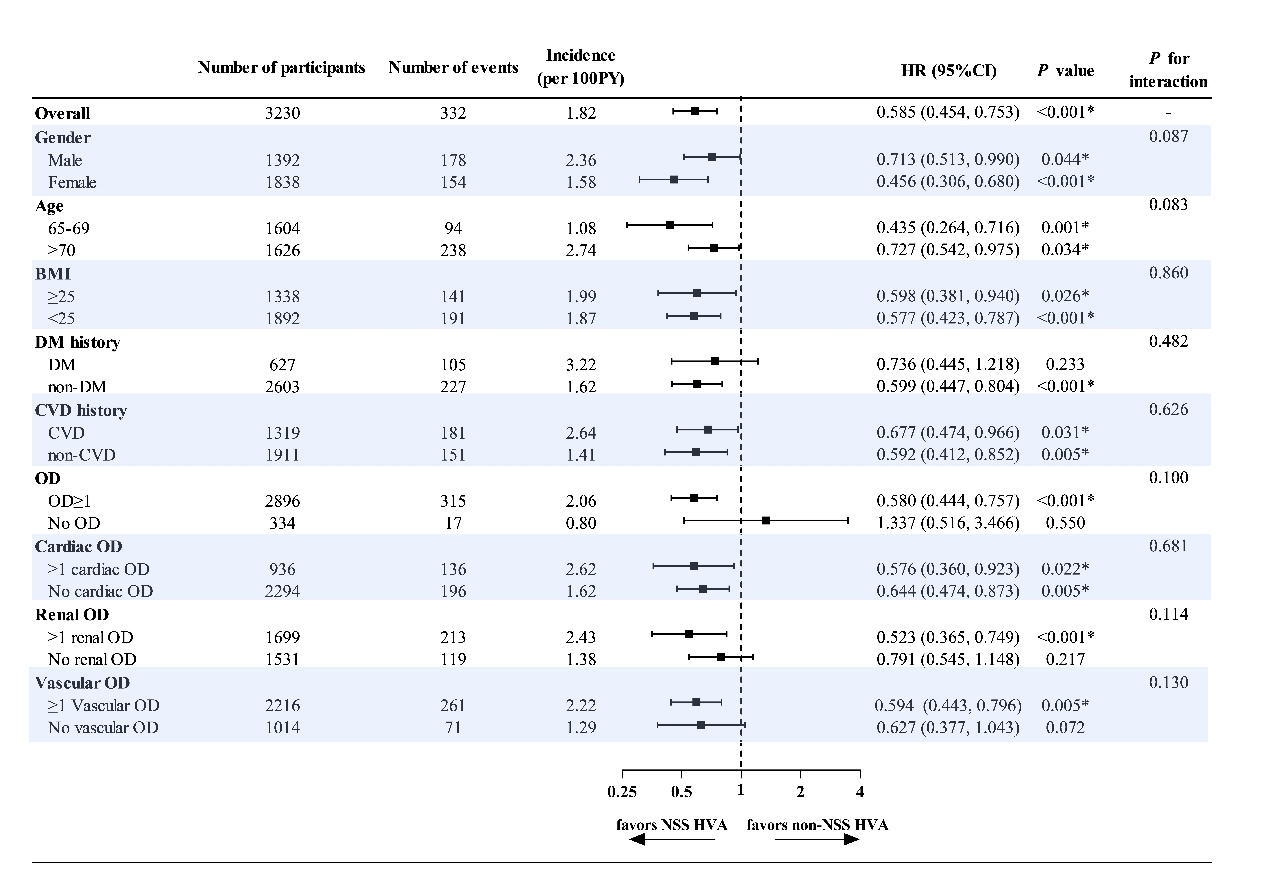
**

**Supplemental figure 1. Forest plot of subgroup analysis for all-cause death.** BMI denotes body mass index, CVD cardiovascular disease, DM diabetes mellitus, OD target organ damage, PY person-year, * indicates significant P value < 0.05.
